# Intersection Intelligence: Supporting Urban Platooning with Virtual Traffic Lights over Virtualized Intersection-Based Routing

**DOI:** 10.3390/s18114054

**Published:** 2018-11-20

**Authors:** José Víctor Saiáns-Vázquez, Esteban Fernando Ordóñez-Morales, Martín López-Nores, Yolanda Blanco-Fernández, Jack Fernando Bravo-Torres, José Juan Pazos-Arias, Alberto Gil-Solla, Manuel Ramos-Cabrer

**Affiliations:** 1 AtlantTIC Research Center, Department of Telematics Engineering, University of Vigo, 36310 Vigo, Spain; vsaians@det.uvigo.es (J.V.S.-V.); yolanda@det.uvigo.es (Y.B.-F.); jose@det.uvigo.es (J.J.P.-A.); agil@det.uvigo.es (A.G.-S.); mramos@det.uvigo.es (M.R.-C.); 2 Research Center on Development and Innovation in Engineering, Universidad Politécnica Salesiana, Cuenca 010102, Ecuador; eordonez@ups.edu.ec (E.F.O.-M.); jbravo@ups.edu.ec (J.F.B.-T.)

**Keywords:** Intelligent Transportation Systems, virtual traffic lights, urban platooning, virtualization, intersection intelligence

## Abstract

The advent of the autonomous car is paving the road to the realization of ideas that will help optimize traffic flows, increase safety and reduce fuel consumption, among other advantages. We present one proposal to bring together Virtual Traffics Lights (VTLs) and platooning in urban scenarios, leaning on vehicle-to-vehicle (V2V) communication protocols that turn intersections into virtual containers of data. Newly-introduced protocols for the combined management of VTLs and platoons are validated by simulation, comparing a range of routing protocols for the vehicular networks with the baseline given by common deployments of traditional traffic lights ruled by state-of-the-art policies. The simulation results show that the combination of VTLs and platoons can achieve significant reductions in travel times and fuel consumption, provided that proper algorithms are used to handle the V2V communications.

## 1. Introduction

Cars are becoming increasingly equipped with sensing, computing and communication capabilities. Fed by advances in many areas of computer science (from signal processing and wireless communications to computer vision, human-computer interaction and many others), technology has been yielding significant advances in terms of safety during the last few years. Advanced Driving Assistance Systems (ADAS) are now commonplace, covering features such as automatic braking, collision protection and emergency assistance [1]. Likewise, better connectivity through vehicle-to-vehicle (V2V) and vehicle-to-infrastructure (V2I) communications [2] enables the automobiles to assemble accurate views of the areas they traverse, and thereby optimize their routes and fuel consumption [3]. Assistance to find parking spaces and richer entertainment services are among recent proposals too [4,5]. However, the major change will be that of autonomous driving, featuring adaptive cruise control, self-parking, highway autopilot and more [6].

In this paper, we investigate a combination of two ideas enabled by V2V communications for the optimization of traffic flow and fuel consumption, leaning on a certain level of automation in the driving:

- On the one hand, we consider virtual traffic lights (VTLs), which were proposed to overcome the inefficiency of the classic traffic light systems by letting the vehicles in the vicinity of an intersection create and coordinate the traffic light signals by themselves [7]. An autonomous driving system can take control of the steering wheel, brake and throttle to pass the intersection safely, auto-adjusting the path and the speed to avoid collisions and maximize the fluency of the traffic.
- On the other hand, we look at *platooning*, a mode of cooperative driving in which groups of vehicles travel as single units (the platoons) for some time, by maintaining a very small and nearly constant distance between them [8,9]. Low-latency communications and autonomous driving features can eliminate the gaps needed for human reaction, increasing road capacity, reducing congestion and achieving greater fuel economy due to reduced air resistance [10,11].

Whereas previous works have dealt with virtual traffic lights that coordinate individual vehicles only, and platooning has been predominantly investigated in highway scenarios, in this paper we present an approach to coordinate groups of vehicles that form and evolve, whenever possible, at the intersections of city roads. Once the platoons are formed, we estimate and disseminate the arrival times to the intersections along their routes. That information is maintained and updated periodically at each intersection, so that any passing vehicles can check whether they might benefit from joining any nearby platoon. The information is also used to adapt the VTLs to facilitate the transit of the platoons.

We present protocols for the management of VTLs and urban platoons, built upon a virtual infrastructure for urban V2V communications [12,13] that turns intersections into virtual containers of data and algorithms that facilitate platoon join maneuvers (hence the title “intersection intelligence”). The proposal is assessed by means of simulations, measuring the performance achieved with various communication protocols in terms of travel duration, fuel consumption and number of platoon join operations completed successfully.

The paper is organized as follows. Section 2 provides a summary of the state-of-the-art in approaches to virtual traffic lights (in cities) and platooning (in highways). Then, Section 3 includes some background information on the virtual communications infrastructure from our previous works, and on the routing protocols we used in the simulations. Section 4 presents the main procedures that we have devised to manage the urban platoons and coordinate the virtual traffic lights. The evaluation study is described in Section 5, with Section 6 discussing the results. Finally, conclusions are given in Section 7, along with the motivation of our ongoing work.

## 2. Related Work

Intersections tend to become the bottlenecks of the traffic flow, especially in the urban environment, where the traffic density is usually greater. Traffic management at the intersections, therefore, takes great importance. However, even in big cities all over the world such as New York, Dublin, or Porto, barely one quarter of the intersections are governed by traffic lights [14]. Furthermore, the operation of the traditional traffic lights systems causes significant delays [15], as they usually work under fixed parametrization rather than adapting to the traffic conditions.

Notable early proposals aimed at improving the effectiveness of the traffic lights relied on Artificial Intelligence, starting from fuzzy approaches. In Ref. [16], for example, a fuzzy logic controller was implemented in an isolated intersection and compared with a conventional controller, achieving better results in terms of average delay of individual vehicles. Taking the proposal one step further, the authors of [17] devised a distributed approach that adapted the signal timing of the traffic lights by managing data about local traffic conditions and the timing of adjacent intersections, which yielded significant reductions in waiting times and number of stops in a Manhattan grid scenario. Other authors have proposed solutions based on Artificial Neural Networks (ANN), addressing the non-linearity characteristics of the vehicular traffic and trying to find an efficient and self-adapting management. An approach based on ANN was presented in [18] that used real-time traffic information to dynamically generate the best timing plan, obtaining improvements in a Manhattan grid of 3 × 3 intersections. When V2V and V2I communications came into scene, a traffic-lights-to-vehicle communication protocol was proposed in [19] reduce fuel consumption, by broadcasting the scheduling information of the traffic lights so that the vehicles could adapt their speed trying to avoid stopping at a red light. These and other approaches can work well around intersections already covered by traffic lights, at the expense of infrastructure investments that could happen anyway as cities around the world turn to smart cities. However, as mentioned above, the percentage of intersections managed by traffic lights is very low, implying that the idea of making the real traffic lights smarter can only have limited impact.

Virtual Traffic Lights promise to remove the dependence on an infrastructure, and therefore bring the benefits of new technologies to every intersection. Taking V2V capabilities for granted, the idea is that the vehicles work together towards self-organizing intersection management, so that the timing of the traffic lights is negotiated according to a certain protocol and disseminated around the intersection in real time. The most relevant proposal was presented in [14], and then refined and field-tested in [20]. In this approach, a vehicle is chosen cooperatively as the VTL leader of an intersection, and from that moment on it is responsible for VTL coordination and dissemination, until it leaves the intersection and a new cycle starts. The simulation results of [14] showed that, in cities with a reduced number of traffic lights, the VTLs can reduce traffic congestion up to 60% at high vehicular densities.

The study of driving patterns has become an issue of great research interest since traffic congestion started to be a problem, already in the first decades of the 20th century [21]. The research community began to perceive the benefits of the platooning of vehicles in the 70s. For example, the author of [22] tried grouping buses together in a platoon to facilitate flow through a central business district in New York during peak hours. However, it was not until the 90s that the first technology-enabled platooning systems appeared. The concept has evolved from approaches with manual steering, in which only the automation in the longitudinal direction was considered [23,24], to more sophisticated ones based on wireless communications and fully autonomous vehicles [25]. As noted above, the latter have largely focused on highway contexts, where the maneuvers for joining and leaving the platoons are quite simple due to the existence of several lanes in each direction—some authors (e.g., [26]) even proposed an additional lane for such maneuvers for simpler management. In practice, one of the most renowned projects is SARTRE (SAfe Road TRains for Environment) [27], which promotes the use of platoons in such a way that several roads are scheduled to travel along the highways, and drivers can join/leave them whenever they want. SARTRE has been successfully tested several times in European roads and their authors claim that they are ready to investigate putting a final product in the market.

Platooning seems to be the most immediate step towards a real Automated Highway System (AHS), but its multiple virtues should be feasible to materialize also in urban scenarios. The authors of [28] showed, by means of simulations, that the throughput of an urban road system can be doubled without changing the signal control, just making the automobiles cross the intersections in platoons rather than one by one with the typical reaction times of human drivers. This hinted to us that combining urban platooning with virtual traffic lights could achieve greater benefits, by introducing the existence of the convoys in the planning of the VTLs and managing join/leave maneuvers in the intersections and all along the road segments between them. Our proposal is to make the idea work with no reliance on any kind of infrastructure, just by means of V2V communications within vehicular ad hoc networks (VANETs). While some authors have considered cloud-based management of VTLs [29] and platoons [30], a solution based on VANETs can be much more responsive, since no data packets must travel to remote servers and back. By avoiding such latencies, we can intertwine computations with communications and keep more fine-grained track of the moves of both individual vehicles and platoons, and deal with any unexpected circumstances more swiftly.

## 3. The Supporting Communication Protocols

**Figure 1:**
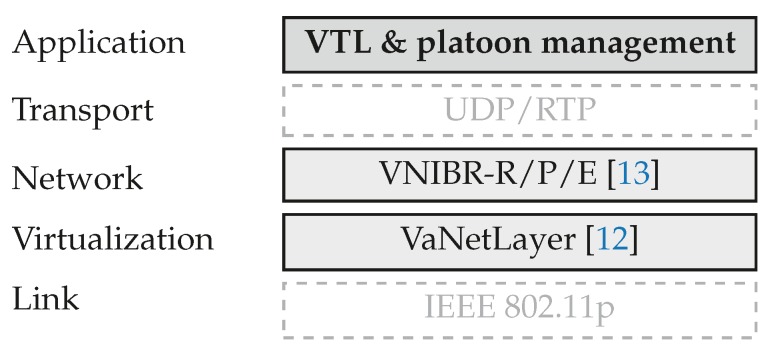
The stack of protocols of our approach.

As noted in Section 1, one of the core ideas of our proposal is to disseminate the arrival times of platoons to the intersections along their routes, and to maintain that information at each intersection. This is a big deal with conventional communication protocols in VANETs, not so much because they have difficulties to tackle the high mobility of the nodes (the literature of VANET routing is now rich and extensive [31]), but because they do not provide the means to maintain persistent information at any given location without the support of external infrastructure. For this reason, our proposal is grounded on a virtualization layer and specific routing algorithms presented in [12,13], adding new mechanisms to manage platoons and VTLs. The stack of protocols we use is represented in Figure 1.

In the following subsections, we summarize the mechanisms than underpin our proposal from the virtualization layer (Section 3.1) and the network layer (Section 3.2). Further details and experimental assessments can be found at [12,13], showing that these underlying protocols can attain good performance in urban scenarios of V2V and V2I communications.

### 3.1. The Virtualization Layer: VaNetLayer

**Figure 2:**
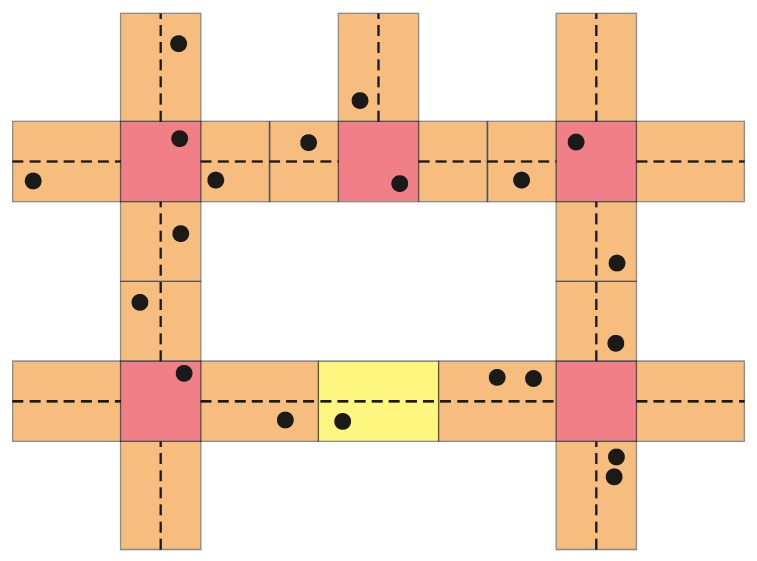
The intersection-based layout of the VaNetLayer (the circles represent vehicles).

The VaNetLayer defines procedures for several vehicles (the physical nodes, PNs) to collaborate in the emulation of stationary virtual nodes (VNs) that can be used to store persistent state information (PSI). As shown in Figure 2, the VNs cover non-overlapping regions, delimited so that there is one VN at each crossroads, and the road segments in between are covered with as many VNs as needed ensure that every PN can communicate directly with all the other PNs in its current region and the adjacent ones.

The main task in the operation of a VN is the distribution of roles of leaders and backups among the PNs. The leader of a region takes charge of packet reception, buffering and forwarding in the communication with other VNs, whereas the backups maintain replicas of the PSI from the virtualization layer and the protocols above. This way, the virtual node can work even when the supporting vehicles fail or leave the region, if there remains at least one inside.

The PSI managed at the virtualization layer (which is the cornerstone of VN emulation) includes counters of nodes and messages from the current region and the neighboring ones, as well as the MAC addresses of their leaders. An exponentially weighted moving average of the number of PNs is computed, too, that provides a basis for QoS estimations.

The interface offered by the VaNetLayer to the protocols above exposes the notion of regions, the role (leader or backup) played by a PN at each moment, and functions to send/receive messages and to get/set/check the PSI. Since the PSI may be crucial for some protocols to run properly, the VaNetLayer implements mechanisms to try to preserve it, even if a region becomes empty for some time (see [12]).

### 3.2. The Virtualized Routing Protocols: VNIBR

The layout of virtual nodes defined by the VaNetLayer provides convenient grounds to develop a combination of topological and geographical routing, with road-based paths connecting successive intersections that lead from sources to destinations. Specifically, VNIBR (Intersection-Based Routing on Virtual Nodes) differentiates three types of routing entities (hence the coloring of Figure 2):

- The level 1 entities (L1VNs, red in Figure 2) cover the regions located at the intersections. L1VNs are the place in which the routing decisions are made, and where the relevant information (e.g., routing tables or lists of vehicle encounters) is kept as PSI.
- The level 2 entities (L2VNs, orange in Figure 2) cover the regions neighboring an intersection. L2VNs forward packets onto a road segment as mandated by the neighboring L1VN, regardless of which vehicle actually does the transmission. Additionally, L2VNs act as backing entities that trying to continue relaying packets onto other road segments during downtimes of the neighboring L1VNs.
- The level 3 entities (L3VNs, yellow in Figure 2) cover the regions in intermediate positions of road segments. The basic task of L3VNs is to relay packets from one end to the other, again regardless of the specific PNs involved.

L1VNs on opposite ends of a road segment exchange *HELLO* packets steadily, to keep track of the connectivity conditions between them. The messages are transmitted hop-by-hop across the L2VNs and L3VNs, which do no other processing than adding the average number of PNs in the corresponding regions. Upon receipt of a *HELLO* packet, an L1VN assigns a QoS value to the road segment, estimating the L1VN-to-L1VN transmission delay, the average number of vehicles in the intermediate virtual nodes, and the number of messages delivered and lost.

On top of these foundations, three different flavors of VNIBR were developed, which we use in the experiments of Section 5: the reactive version (VNIBR-R) builds the communication routes on request, looking for the destination by broadcasting requests omnidirectionally; the proactive version (VNIBR-P) manages trees of routes between intersections, aiming to provide faster responses at the expense of increased overhead; finally, the encounter-based version (VNIBR-E) builds the routes on demand, but aided by lists that keep track of the last time any vehicle traversed any intersection. The details of the operation of the three protocols can be found in [13].

## 4. Procedures for VTL and Platoon Management over the Virtual Nodes

The following subsections present the main procedures we have defined to manage virtual traffic lights and platoons, relying on the communication facilities enabled by the infrastructure of virtual nodes. These have to do with (i) the dissemination of the expected times of platoon arrivals at the intersections, and (ii) the selection of a vehicle to act as VTL leader and control the virtual signaling, and (iii) the VTL computations coupled with the management of platooning maneuvers.

### 4.1. Platooning Announcement on Virtual Nodes

It is unlikely that the vehicles that might be interested in joining a certain platoon ( reach some place in an efficient and comfortable way) will just come across it. Therefore, it is important to disseminate information about existing platoons, their destinations, and the expected times of arrival at different locations along their routes, so that any vehicles can make informed decisions on whether to join a platoon, adjusting its own timing and even the routes they planned to follow accordingly. The dissemination of information is also central in our approach, since it affects the timing of the VTLs and, ultimately, proper global behavior depends on having a reasonably cohesive and updated view of the vehicular traffic at the L1VNs (the virtual nodes at the intersections).

In our approach, the platoon leaders (the vehicles at the head of the trains) periodically send announcement messages that make their way through the sequences of L1VNs along the routes they are going to follow. Those L1VNs thus learn about the current length, position, and route of the platoon. Thereby, they can update the PSI of the corresponding intersection, with entries containing a timestamp, platoon ID, an estimated times of arrival of the platoon to successive intersections. The estimations are made according to an exponentially weighted moving average (EWMA) of the transit times along the road segments, which are readily measured by the VaNetLayer and VNIBR down in the protocol stack, for the two possible directions (If no data is received for some road segment for a relatively long time, the L1VN assumes that it is empty and the transit time is estimated from the length and a speed close to the maximum allowed). An estimation of the time taken to go through the intersections is added, too, taking into account that platoons will be given priority over individual vehicles by the VTLs.

Since the accuracy of the estimations inevitably degrade in each hop between L1CNs, each estimation in the PSI is labeled with a value between 1 and 4 that represents the amount of uncertainty in the estimation. Accordingly, the announcement messages are only forwarded to the next 4 intersections on the route of the platoon, and each L1VN in turn disseminates the estimations towards nearby intersections (also up to 4 road segments away).

**Figure 3:**
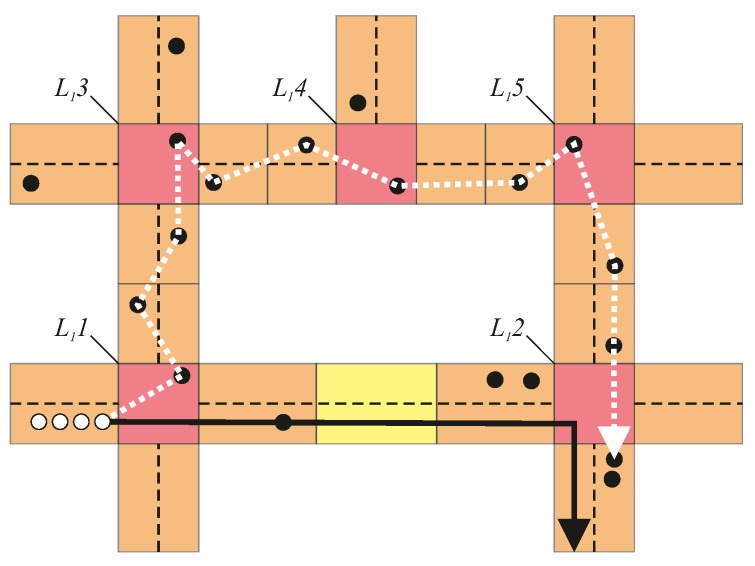
Platoon announcement along the L1VNs in the platoon’s route.

It is worth noting that, as shown in Figure 3, the route followed in the dissemination of the announcement message (represented by a dotted white line) does not necessarily match the platoon’s route (the solid black line). The data packets will be forwarded passing through all the L1VNs in the platoon’s route, but following the routes decided by the VNIBR protocol. In the example, the platoon leader sends the announcement to the next L1VN in its route (lower left corner). As mandated by the routing protocol, the next hop is $L_{1}2$; however, since $L_{1}1$ senses poor QoS over the road segment to $L_{1}2$ (due to the large gap of the empty L3VN between $L_{1}1$ and $L_{1}2$), the packets are delivered via a detour through $L_{1}3$, $L_{1}4$ and $L_{1}5$.

### 4.2. VTL Leader Election

In the pioneering works of [14,32], the operation of a VTL requires one of the vehicles approaching an intersection to be chosen as the VTL leader. From that moment on, that vehicle is responsible for VTL coordination and dissemination, until it leaves the intersection and a new cycle starts. Since we rely on a network of virtual nodes placed around each intersection, it is straightforward to decide which vehicles are candidates for the role of VTL leader. Hereafter, we will use the term “*VTL area*” to mean the aggregate of the regions covered by the L1VN placed at a given intersection and the L2VNs adjacent to it. The inclusion of the L2VNs is intended to start the VTL processes early enough to get the VTL information before arriving to the intersection.

The process to grant the role of leader/non-leader to each one of the PNs in the VTL area is driven by a finite state machine controlled by events of message receipts, timeouts and region changes. Each PN can be in one out of the six following states:

- *OUT OF INTERSECTION*: the PN is not taking part in the VTL action.
- *REQUEST*: the PN is requesting VTL leadership.
- *ASCERTAINMENT*: this is an intermediate state, needed to avoid problems of duplicate leadership that would result in contradictory VTL signals (The problem of duplicate leadership was analyzed in [32], concluding that VTLs are still feasible if it occurs from time to time. We preferred minimizing the probability, at the expense of a few milliseconds in the VTL operation).
- *LEADER*: the PN is VTL leader for the intersection.
- *NOT LEADER*: the PN is waiting for the VTL leader to inform of VTL schedule.
- *GREEN LIGHT*: the PN has received the green light and aims to cross the intersection.

The protocol defines eight types of messages:

- *M_LeaderRequest*: used to request leadership.
- *M_LeaderResponse*: sent by a leader to decline a leader request or a leader ascertainment, and by a PN in the *ASCERTAINMENT* state to reply to another PNs that reached that state later.
- *M_VTL*: used by the leader to periodically disseminate the VTL schedule.
- *M_LeaderAnnouncement*: used by a PN in the *ASCERTAINMENT* state to announce that it is going to become VTL leader.
- *M_IntersectionLeft*: sent by any vehicle leaving an intersection.
- *M_LeaderDesignation*: sent by a leader when it finally gets the green light, to start the next leader election cycle. The leader can fill in a given field in this message to indicate the ID of a candidate to become the next leader (a PN that is known to stay within the VTL area long enough) to speed up the process.
- *M_CancelVTL*: used by a PN that accidentally assumed leadership after realizing there was a standing leader; the message reports the incident to the vehicles that received the green light in the last *M_VTL* from the duplicate leader.
- *M_Hello*: sent periodically by every vehicle since it enters the VTL area, indicating its distance to the intersection, its intended path, and whether it is interested in joining a given platoon.

There are also four different timers:

- *T_RequestWait*: used by a PN in the *REQUEST* state to discard the existence of a standing VTL leader after sending an *M_LeaderRequest* message.
- *T_Ascertainment*: used by a PN in the *ASCERTAINMENT* state to discard the existence of a standing VTL leader after sending an *M_LeaderAnnouncement* message.
- *T_VTLWait*: used by leaders and non-leaders to control when to send or expect *M_VTL* messages.
- *T_Hello*: used by every PN to control when to send *M_Hello* messages.

**Figure 4:**
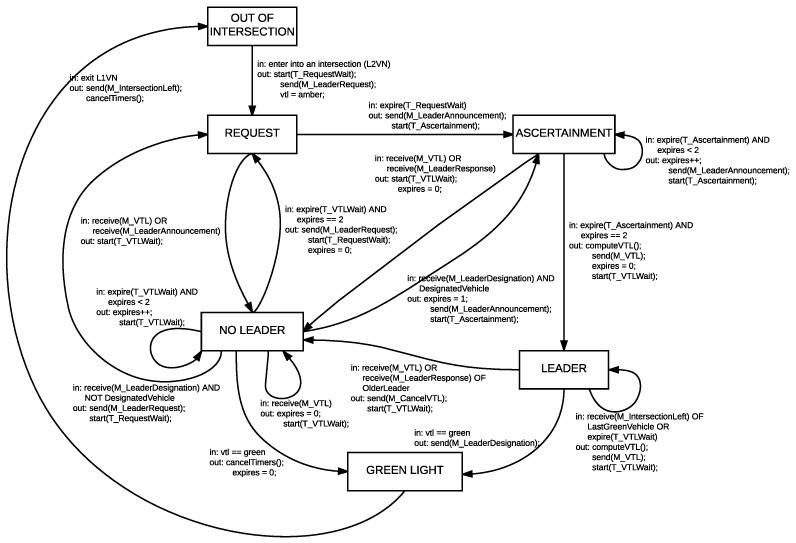
The state machine of the procedure used to choose a VTL leader.

The behavior of the protocol is represented in the graph of Figure 4:

- Every vehicle starts in the *OUT OF INTERSECTION* state. When it enters the VTL area, it switches to *REQUEST*, changes its VTL signal to amber and broadcasts an *M_LeaderRequest* message, starting the *T_RequestWait* timer. If it receives *M_VTL* or *M_LeaderAnnouncement*, the PN switches to *NOT LEADER* and changes its VTL signal according to the leader instructions; otherwise, it transitions into the *ASCERTAINMENT* state and broadcasts *M_LeaderAnnouncement*.
- From the *ASCERTAINMENT* state, the PN waits twice for confirmation of its leadership during the time defined by *T_Ascertainment*, re-broadcasting the *M_LeaderAnnouncement* after the first expiration. If it receives a message before *T_Ascertainment* expires twice (The message may be *M_VTL* or *M_LeaderResponse*, coming from the current leader, or *M_LeaderAnnouncement*, coming from other nodes that reached the *ASCERTAINMENT* state before), its state changes to *NOT LEADER*; otherwise, the PN is confirmed as leader and transitions into *LEADER* state. If an *M_VTL* or *M_LeaderResponse* message tells it about an earlier leader afterwards, the PN switches to *NOT LEADER* and broadcasts an *M_CancelVTL* message.
- When a vehicle in *LEADER* state gets the green light and proceeds to cross the intersection, it switches to *GREEN LIGHT* state and broadcasts *M_LeaderDesignation* to trigger a new leader designation process. This takes all the other nodes to *REQUEST* state, except—if present—the designated vehicle announced by the leader, which jumps directly to the second stage of the *ASCERTAINMENT* state to speed up the new election.
- A PN in *NOT LEADER* state uses *T_VTLWait* to detect the presence of a leader. The timer is restarted upon receipt of *M_VTL*. If it expires twice, the PN assumes the former leader’s withdrawal and switches to *REQUEST* state.
- When a PN exits the L1VN after receiving the green light, it sends an *M_IntersectionLeft* message, switching to the initial state *OUT OF INTERSECTION*.

### 4.3. VTL Computation and Platooning Maneuvers

Once a VTL leader has been elected, it proceeds to compute and disseminate the VTL signals in the VTL area. For this purpose, it maintains a table of information –computed from the *M_Hello* messages—about the vehicles arriving at the intersection through any of the neighboring L2VNs. According to this information, in a general case, the leader will give the green light to the first vehicles in the most congested lane, as well as the first vehicles in other inlets, as long as their trajectories do not interfere. The rest of the lanes will be served sequentially, always giving the green light to several vehicles proportional to the number of vehicles detected in each lane. This way, we try to avoid congestion in the busiest roads without incurring in starvation at the least busy ones. When the cycle finishes and the leader receives the green light, it sends an *M_LeaderDesignation* message to start the new leader election.

Up to here, the VTL operation is largely similar to the proposal of [14], with priorities assigned depending on congestion measurements. Our approach, however, makes some additional provisions to facilitate the flow of the platoons and vehicles interested in joining them, taking advantage from the fact that the platooning announcements are processed and leave persistent information in the L1VNs:

- On the one hand, the VTL leaders check that information so that, when a platoon is about to arrive at the intersection, the arrival lane gets increased priority, giving the green light to a greater number of vehicles in every cycle (until the estimation time plus a little margin of error that depends on the distance of the platoon since the last announcement).
- On the other hand, vehicles interested in joining a platoon can check the estimated arrivals information and try to reach the intersection on time. The potential joiners include the ID of the platoon in their *M_Hello* messages, so the VTL leader can give green lights selectively to facilitate their incorporation at the end of the trains.

**Figure 5:**
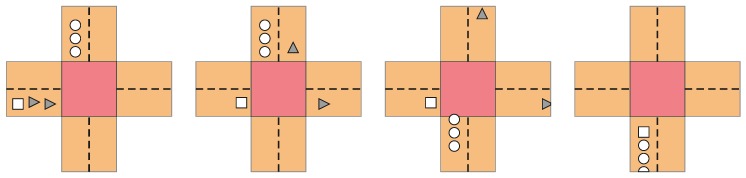
Join maneuver coordinated by the VTL protocol.

A join maneuver is depicted in Figure 5. The car represented by the white square wants to join the platoon led by the one represented by a white circle, but two other vehicles ahead of it—represented by gray triangles—obstruct the operation. In this situation, the VTL leader gives the green light to those two vehicles first, then to the platoon, and finally to the joiner. It must be noticed that our algorithm follows a ”first come, first served” policy with the potential joiners, as there may be situations in which more than one vehicle is ready to join the platoon, but not all of them can be satisfied due to the traffic conditions.

Currently, the join maneuvers are only considered when the potential joiners are awaiting at the intersection upon arrival of the platoon. If a potential joiner arrives to the intersection before the platoon, it is treated as an ordinary vehicle, so it may green light before the arrival of the platoon. Therefore, the joining does not happen—it could, afterwards, if the platoon arrives from behind and the vehicle becomes the head of it. We do not consider delaying the traffic flow along the lane of early-arriving vehicles, because it could increase congestion and make other vehicles wait unnecessarily to cross the intersection.

Leave maneuvers are easier to handle, because there is no need for coordination by the VTL leader. Yet, the intended path of the vehicle that wants to leave a platoon must be notified to the VTL leader in the *M_Hello* messages, to avoid giving at the same time the green light to other vehicles that could interfere with it. The leaving vehicle only must communicate with the platoon leader to increase the space with its preceding and following vehicles as soon as the platoon receives the green light.

## 5. Experimental Evaluation

**Figure 6:**
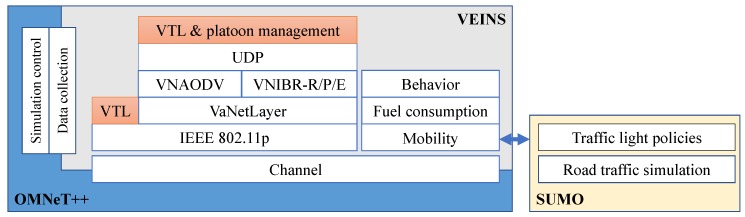
Architecture of our VEINS-based simulator.

We have assessed the benefits of our proposed combination of VTLs and urban platoons via simulation. The scenario consisted of a Manhattan grid urban environment of 7 × 7 intersections and 1375 × 1375 m with two lanes per road (one in each direction) and a maximum speed of 50 Km/h. To simulate an application in which the exchange of information among the vehicles may affect their movements, we resorted to VEINS [33], a tool that combines an event-based network simulator (OMNeT++) and a road traffic simulator (SUMO). The architecture, including the modules from our own works, is shown in Figure 6.

Our goal was to compare the performance of four different configurations:

- **Configuration 0 (traditional traffic lights only)** provides the performance baseline, managing no platoons and no VTLs. Rather, there are 12 intersections governed by traditional traffic lights (Twelve intersections is nearly one quarter, in line with what we explained is common for big cities in Section 2). We implemented a SUMO module to provide three different policies for these traffic lights: a fixed-time policy (FT), the *Max-Pressure* (MP) feedback control of [34] and the *Adaptive Max-Pressure* (AMP) of [35]. Their operation was completely autonomous, not informed of the existence of platoons or any other communications.
- **Configuration 1 (One-hop VTLs)** is representative of the state-of-the-art, with no platoon management, but with VTLs running in each intersection according to the algorithm proposed in [14], with the implementation and parameterization of [20]. All communications among the vehicles are one-hop, implemented directly on top of IEEE 802.11p.
- **Configuration 2 (traditional traffic lights + platoons)** incorporates our proposal for platoon management only, with traditional traffic lights as in configuration 0. Nearby vehicles steadily exchange information about their routes (via one-hop broadcasting), and a platoon is formed (and announced in multiple hops, as presented in Section 4.1) wherever two consecutive cars find that they will be one in front of the other for 3 subsequent intersections or more. Any other vehicles consider joining a platoon if they will be in it for 2 road segments or more, but no one considers deviations from its intended route.
- **Configuration 3 (VTLs + platoons)** represents the complete proposal of this paper, with the same platoon joining policy of configuration 2 and the platoon-aware VTL management procedures of Section 4.2 and Section 4.3.

For configurations 2 and 3, we developed the VTL & platoon management procedures within VEINS, and reused implementations of the VaNetLayer and VNIBR protocols as OMNeT++ modules (The space between intersections in the simulation scenario allowed two L2VNs and one L3VN along each road segment). The *Platooning Extension for VEINS* [36] was used to implement the platooning operations, with precise control on the vehicles’ speeds and distances. As an alternative to VNIBR for routing, we wrote an OMNeT++ implementation of VNAODV (*Ad hoc On-Demand Distance Vector on Virtual Nodes*), an algorithm proposed in [37] to demonstrate the capabilities of the virtualization layer that preceded the VaNetLayer (see [38]). The comparison between VNAODV and the different flavors of VNIBR serves to highlight the gains from a purely intersection-based routing approach.

We ran simulations of configurations 0, 1, 2 and 3 with three different vehicular densities: low (512 vehicles), medium (1024 vehicles) and high (2048 vehicles). The sets of routes for the vehicles were generated randomly, but each set would be used under the four configurations to ensure fair comparisons. Within these settings, we were interested in measuring travel times and fuel consumption for all the vehicles, as well as for those that had been in a platoon (at the head of it or not) at least once. The fuel consumption estimations were made according to the HBEFA 3 model [39]. We also looked at the average number of cars in the platoons, as an indicator of how much the join maneuvers were facilitated.

The results are presented in Table 1, Table 2 and Table 3 for low, medium and high traffic densities, respectively. To facilitate their interpretation, we directly present the average values of travel times and fuel consumption from configurations 1, 2 and 3 normalized with regard to the best average values from configuration 0 (that is, choosing the most favorable case for traditional traffic lights from the results achieved by FT, MP or AMP policies).

## 6. Discussion

The first observation from the results is that the VTL mechanisms (both the algorithm of [14,20] and ours) make a major difference by themselves, inasmuch as travel times and fuel consumption are significantly lower in configurations 1 and 3 than in configurations 0 and 2, notwithstanding which policy drives the traditional traffic lights in the latter. Global savings of around 5% in travel times and around 10% in fuel consumption are, undoubtedly, great incentives to develop standard VTL protocols, to be supported by the majority of the vehicles.

In the comparison between configurations 1 and 3, it turns out that our proposal for VTL management attains greater savings than that of [14,20]. The average difference is around 2% of global travel times and 3% of fuel consumption, which can be taken as a justification for the increased complexity of our stack of protocols (Figure 1) in comparison with placing VTL logic directly on top of IEEE 802.11p (It must be noted that the VaNetLayer and VNIBR protocols are of general use, so they can support any other communication services in VANETs in addition to VTL management, as explained in [12,13]). The advantages are most noticeable with medium traffic densities, because our proposal—thanks to the virtualization infrastructure—supports multi-hop communications and persistent state information; therefore, we can manage larger VTL areas to coordinate more incoming vehicles at each intersection. The difference is not so noticeable with light traffic, because the vehicles can go through the crossroads swiftly most of the times, and so any VTL logic can work well even if some messages are lost. With heavy traffic, in turn, vehicle movements are slow enough so that more efficient communications do not bring about better VTL performance.

On the other hand, we can see that platooning alone can attain travel time reductions of up to 7% under light traffic conditions, and up to 2% with heavy traffic (the aerodynamic advantages of travelling very close to the preceding car vanish when the average speed is low). However, when platoons are combined with VTLs that seek to facilitate their transit through every intersection (which is only possible with our proposal), the savings go from nearly 65% with light traffic to almost 72% with heavy traffic: there is more to gain when congestion is not an issue after each road segment. Anyway, the benefit is substantial, and it is not achieved to the detriment of cars that do not participate in platoons, as evidenced by the fact that the global averages are better in configuration 2 than in configuration 0, and better in configuration 3 than in configuration 1 as well.

Looking at the figures of configuration 3 in Table 1, Table 2 and Table 3, it turns out that the combined management of VTLs and platoons is most beneficial for platoon members in cases of high traffic density, but the global savings are greater with medium density. The reason is that, in the latter case, it is easier for the VTL logic to match an incoming platoon and one or more cars interested in joining them. In congested scenarios, in contrast, those cars may still be queueing behind a few others when the platoon crosses the intersection.

Our proposal for VTL management also has noticeable impact in the average length of the platoons, because it promotes the join maneuvers. Without VTLs, in configuration 2, the average length of the platoons is close to the minimum (2) and it is common that an existing platoon is split at an intersection, since not all the members manage to cross according to the traditional traffic lights or the stop/yield signals.

Finally, it can be seen that the underlying communication protocols have noticeable impact in the performance of configuration 3 and, to a lesser extent, configuration 2 too. VNAODV yields the worst performance systematically, because it stores and processes routing information in every virtual node (not only at the intersections), which implies slower action and more possible points of failure. These shortcomings are more noticeable with light traffic, when there are fewer cars in general to support every virtual node. Among the three variants of VNIBR, the proactive one achieves the best results with medium and high vehicular densities, because greater stability of the virtual nodes and lower mobility of the vehicles lead to a more usable tree of routes between intersections, which speeds up the dissemination of platoon announcements and helps to increase the accuracy of the arrival estimations. In cases of low vehicular density, the reactive and encounter-based versions get better results, since VNIBR-P has more trouble to deal with the high mobility and the frequent QoS changes. In any case, it is worth noting that, whereas other communication services supported by the infrastructure of virtual nodes could be severely affected by frequently empty VN regions (see [12,13]), the management of VTLs and platoons is quite resilient, because the information exchanged among the vehicles is only relevant in their vicinity, and empty VN regions (particularly, empty intersections) mean faster transit for both individual cars and platoons.

## 7. Conclusions and Future Work

We have presented one approach to the combined use of virtual traffic lights and urban platoons, which may be easy to realize in the short term thanks to the advances in the areas of connected cars and autonomous driving. Our proposal tries to bring the benefits of platooning to the urban environment, managing maneuvers at the intersections with the help of VTLs and promoting new vehicles to join the platoons by disseminating notifications of arrival along their routes.

By means of simulation experiments, we have shown that VTLs and platooning entail the potential to achieve substantial savings in travel times and fuel consumption, not only benefitting the vehicles that participate in platoons, but also improving the overall fluency of the traffic. In contrast, unlike what happens in highways, deploying platooning mechanisms alone with traditional signaling would not yield significant advantages, because the size and the dynamics of city roads do not give many opportunities for platoon join maneuvers—they must be sought and facilitated at the intersections, as indicated by our concept of “*intersection intelligence*”. We have also seen that the extensive coordination among vehicles can be attained with vehicle-to-vehicle communications only (i.e., without resorting to the infrastructure of cellular networks as in [29]) thanks to the good performance of the VaNetLayer and VNIBR protocols.

As part of our ongoing work, we are investigating possible extensions of the proposed VTL & platoon management logic to work with several priority levels. This would allow, for example, ensuring green lights systematically to emergency vehicles. Likewise, public transport, electric, hybrid and high occupancy vehicles could have greater priority levels than regular cars, and they would be favored if the VTL protocol looked at the priority sums in each lane rather than the number of vehicles. We are also seeking to formalize strategies for the integration of the platoon announcement system with GPS navigators, so that the vehicles can modify the routes they intended to follow to enjoy more efficient and comfortable rides. Finally, we are working to categorize and resolve the potential security breaches of our protocols, from the virtualization to the application layer, to prevent misuses and malicious network attacks that could lead to safety threats. We believe that the blockchain architecture presented in [40] can be a good framework for a cohesive solution, that could actually benefit from the infrastructure of virtual nodes that underpins our work.

## Figures and Tables

**Table 1:** Results for low traffic density (time and fuel normalized regarding best average values from configuration 0).

		All Cars		Platoon Members		
		Travel Time	Fuel Consum.	Travel Time	Fuel Consum.	Avg. Platoon Length
Configuration 1 (One-hop VTLs)		0.961	0.914	N/A	N/A	N/A
Configuration 2 (Traditional traffic lights + platoons)	VNIBR-R	0.996	0.993	0.931	0.947	2.18
	VNIBR-P	0.997	0.994	0.939	0.945	2.11
	VNIBR-E	0.996	0.994	0.933	0.949	2.13
	VNAODV	0.999	0.997	0.941	0.951	2.04
Configuration 3 (VTLs + platoons)	VNIBR-R	0.933	0.878	0.364	0.401	3.14
	VNIBR-P	0.954	0.886	0.445	0.496	3.22
	VNIBR-E	0.941	0.882	0.397	0.427	3.11
	VNAODV	0.968	0.913	0.686	0.740	2.64

**Table 2:** Results for medium traffic density (time and fuel normalized regarding best average values from configuration 0).

		All Cars		Platoon Members		
		Travel Time	Fuel Consum.	Travel Time	Fuel Consum.	Avg. Platoon Length
Configuration 1 (One-hop VTLs)		0.947	0.904	N/A	N/A	N/A
Configuration 2 (Traditional traffic lights + platoons)	VNIBR-R	0.998	0.997	0.954	0.960	2.21
	VNIBR-P	0.997	0.996	0.947	0.971	2.28
	VNIBR-E	0.998	0.997	0.956	0.962	2.18
	VNAODV	0.999	0.998	0.976	0.981	2.09
Configuration 3 (VTLs + platoons)	VNIBR-R	0.918	0.861	0.357	0.402	4.15
	VNIBR-P	0.909	0.870	0.257	0.286	4.88
	VNIBR-E	0.914	0.867	0.313	0.252	4.45
	VNAODV	0.932	0.889	0.485	0.506	3.25

**Table 3:** Results for high traffic density (time and fuel normalized regarding best average values from configuration 0).

		All Cars		Platoon Members		
		Travel Time	Fuel Consum.	Travel Time	Fuel Consum.	Avg. Platoon Length
Configuration 1 (One-hop VTLs)		0.943	0.872	N/A	N/A	N/A
Configuration 2 (Traditional traffic lights + platoons)	VNIBR-R	0.999	0.998	0.987	0.991	2.19
	VNIBR-P	0.998	0.997	0.981	0.991	2.26
	VNIBR-E	0.998	0.997	0.989	0.992	2.13
	VNAODV	0.999	0.999	0.994	0.995	2.07
Configuration 3 (VTLs + platoons)	VNIBR-R	0.931	0.851	0.272	0.329	3.54
	VNIBR-P	0.950	0.878	0.138	0.198	3.77
	VNIBR-E	0.937	0.877	0.224	0.251	3.61
	VNAODV	0.944	0.880	0.319	0.362	3.15

## Abbreviations

The following abbreviations are used in this manuscript:

V2V	Vehicle-to-Vehicle
V2I	Vehicle-to-Infrastructure
VANET	Vehicular Ad-hoc Network
PN	Physical Node
VN	Virtual Node
PSI	Persistent State Information
VTL	Virtual Traffic Lights
VNAODV	Ad hoc On-Demand Distance Vector on Virtual Nodes
VNIBR-R	Intersection-Based Routing on Virtual Nodes—Reactive
VNIBR-P	Intersection-Based Routing on Virtual Nodes—Proactive
VNIBR-E	Intersection-Based Routing on Virtual Nodes—Encounter-based
L1VN	Level 1 Virtual Node
L2VN	Level 2 Virtual Node
L3VN	Level 3 Virtual Node
FT	Fixed-time
MP	Max-Pressure
AMP	Adaptive Max-Pressure
GPS	Global Positioning System
